# Key Learnings During the Development of a Generic Data Collection Tool to Support Assessment of Freedom of Infection in Cattle Herds

**DOI:** 10.3389/fvets.2021.656336

**Published:** 2021-04-26

**Authors:** Annika M. van Roon, Egle Rapaliute, Xhelil Koleci, Violeta Muñoz, Mathilde Mercat, Céline Faverjon, Inge M. G. A. Santman-Berends, Mirjam Nielen, Simon J. More, David Graham, Maria Guelbenzu-Gonzalo, Aurélien Madouasse, Christine Fourichon, Gerdien van Schaik

**Affiliations:** ^1^Unit Farm Animal Health, Department of Population Health Sciences, Faculty of Veterinary Medicine, Utrecht University, Utrecht, Netherlands; ^2^Department of Veterinary Pathobiology, Faculty of Veterinary Medicine, Lithuanian University of Health Sciences, Kaunas, Lithuania; ^3^Faculty of Veterinary Medicine, Agricultural University of Tirana, Tirana, Albania; ^4^SAFOSO AG, Liebefeld, Switzerland; ^5^INRAE, Oniris, BIOEPAR, Nantes, France; ^6^Ausvet Europe, Lyon, France; ^7^Royal GD, Deventer, Netherlands; ^8^Centre for Veterinary Epidemiology and Risk Analysis, Veterinary Sciences Centre, University College Dublin, Dublin, Ireland; ^9^Animal Health Ireland, Carrick-on-Shannon, Ireland

**Keywords:** data collection, output-based, control programmes, freedom from disease, cattle, sound control

## Abstract

Various European Member States have implemented control or eradication programmes for endemic infectious diseases in cattle. The design of these programmes varies between countries and therefore comparison of the outputs of different control programmes is complex. Although output-based methods to estimate the confidence of freedom resulting from these programmes are under development, as yet there is no practical modeling framework applicable to a variety of infectious diseases. Therefore, a data collection tool was developed to evaluate data availability and quality and to collect actual input data required for such a modeling framework. The aim of the current paper is to present the key learnings from the process of the development of this data collection tool. The data collection tool was developed by experts from two international projects: STOC free (Surveillance Tool for Outcome-based Comparison of FREEdom from infection, www.stocfree.eu) and SOUND control (Standardizing OUtput-based surveillance to control Non-regulated Diseases of cattle in the EU, www.sound-control.eu). Initially a data collection tool was developed for assessment of freedom of bovine viral diarrhea virus in six Western European countries. This tool was then further generalized to enable inclusion of data for other cattle diseases i.e., infectious bovine rhinotracheitis and Johne's disease. Subsequently, the tool was pilot-tested by a Western and Eastern European country, discussed with animal health experts from 32 different European countries and further developed for use throughout Europe. The developed online data collection tool includes a wide range of variables that could reasonably influence confidence of freedom, including those relating to cattle demographics, risk factors for introduction and characteristics of disease control programmes. Our results highlight the fact that data requirements for different cattle diseases can be generalized and easily included in a data collection tool. However, there are large differences in data availability and comparability across European countries, presenting challenges to the development of a standardized data collection tool and modeling framework. These key learnings are important for development of any generic data collection tool for animal disease control purposes. Further, the results can facilitate development of output-based modeling frameworks that aim to calculate confidence of freedom from disease.

## Introduction

Surveillance and control of cattle diseases in Europe is essential to protect human and animal health and to facilitate safe trade between member states. This is supported by the Animal Health Law adopted in March 2016. Within the Animal Health Law (EU 2016/429), diseases are listed and categorized (A, B, C, D or E) according to their relevancy for Union intervention (EU 2018/1882). This relevancy depends on their impact on public or animal health, the economy, society or the environment. Diseases listed as category A or B must be eradicated by all Member States and therefore mandatory requirements are legislated within the European Union (EU). Examples of category A or B cattle diseases are foot and mouth disease and Bluetongue. For diseases listed as category C, D, or E, there are only few or no mandatory requirements legislated within the EU (referred to as non-regulated diseases in the remainder of this paper). Examples of non-regulated diseases include bovine viral diarrhea (BVD), infectious bovine rhinotracheitis (IBR) and Johne's disease (JD). Numerous countries in Europe have implemented control programmes (CPs) for these so-called non-regulated cattle diseases. The CPs aim to eradicate, control or monitor infectious diseases in the cattle population. Although these diseases are not regulated by the EU, these CPs are beneficial for farmers, the industry, and national economy as they increase animal health and welfare and reduce direct losses (e.g., production loss, morbidity, and mortality) as well as indirect losses (e.g., constraints to trade) (1). Each country develops CPs to fit their specific situation, e.g., infection status and cattle demographics, and therefore these are very heterogeneous between countries, which is for example the case for BVD (2). This variety causes difficulties for intra-community trade as the outcomes of these CPs are difficult to compare. For example, the confidence that herds deemed to be free from specified infections by a given CP are truly free from infection, and the uncertainty associated with this, may vary between CPs. There are methods, such as scenario tree analysis and Bayesian latent class modeling, that can be used to estimate the confidence of freedom resulting from CPs. However, a transparent, standardized and practical field-based tool is not yet available (3–5).

Two projects were started to fill this gap: the STOC free project (Surveillance Tool for Outcome-based Comparison of FREEdom from infection, www.stocfree.eu) (6) and the COST action SOUND control (Standardizing OUtput-based surveillance to control Non-regulated Diseases of cattle in the EU, www.sound-control.eu) (1, 7). The STOC free project aims to develop an output-based framework to compare the probability of freedom from infection for herds (or animals) assigned an infection-free status in heterogeneous CPs. In this project, partners from six European countries (Germany, France, Ireland, the Netherlands, Sweden, and Scotland) have worked together to develop a framework consisting of a model to calculate the confidence of freedom for the case disease bovine viral diarrhea (BVD) and a data collection tool to collect the data needed to run the model. The aim of SOUND control is to stimulate initiatives to explore innovative methods to substantiate confidence of freedom from infection and describe requirements for an objective and standardized output-based framework for several non-regulated cattle diseases in Europe. In this COST Action, more than 100 researchers from 32 countries collaborate. Both projects have the ultimate aim to develop a set of tools, which also includes a generic data collection tool that can be used by different countries with different CPs to collect the data that are needed for the assessment of confidence of freedom. This is challenging because data are collected, stored and interpreted in different ways in different countries. As an example, national BVD eradication programmes can differ substantially in their approaches to data management and interpretation (2). The same was earlier described for IBR (8). Therefore, consensus is needed on both the data required, and the definitions of these data, to allow assessment of confidence of freedom. In existing methods aimed at demonstrating freedom from disease such as scenario tree modeling, the sensitivity of each surveillance component is assessed by including data on test sensitivity and frequency, the number of herds and animals present and tested within the cattle population, the expected prevalence, and risk factors for infection (5). Further, information is needed on what data are available in different countries and the comparability of these data. The latter is, amongst others, influenced by the quality of the available data (9), which in turn is most commonly assessed based on its completeness, accuracy and timeliness (10).

Tools have been developed to assist in designing CPs, support decision-making and implementation of control strategies. Example include the RISKSUR (Risk-based animal health surveillance systems) project in which decision support tools were developed to assist in the design of surveillance programmes (11) and the HOTLINE (Harmonization Of Transmissible disease Interpretation in the EU) project which sought to make disease information from different countries comparable and interpretable (12). As part of this latter project, guidelines were developed for the reporting of animal health surveillance (AHSURED: Animal Health Surveillance Reporting Guidelines) (13). A list of key surveillance items, such as geographical area, susceptible population, historical situation etc., has been published to guide the reporting of surveillance activities, such as confidence of freedom from infection or prevalence estimation (https://github.com/SVA-SE/AHSURED/wiki). Another project that has common ground with STOC free and SOUND control is the SIGMA project that aims to harmonize data models and automate the process of data submission, validation, analysis, and reporting of EU member states to EFSA (14). These projects are very valuable and have aspects relating to our goal, which is comparison of the outputs of CPs. However, in our project we do not aim to harmonize the input but rather to investigate ways to compare heterogeneous input and generate homogeneous output.

Our objective was to develop a simple and practical online data collection tool that could act as part of an output-based framework that is seeking to model freedom from infection of cattle diseases in different countries. The data collection tool was initially developed for BVD, IBR, and JD. These three diseases were selected because there are many different CPs within Europe (1) and they differ in terms of disease transmission dynamics, accuracy of diagnostic methods etc. The aim of this paper is to present the key learnings from the process of the development of the online data collection tool.

## Materials and Methods

**Figure 1:**
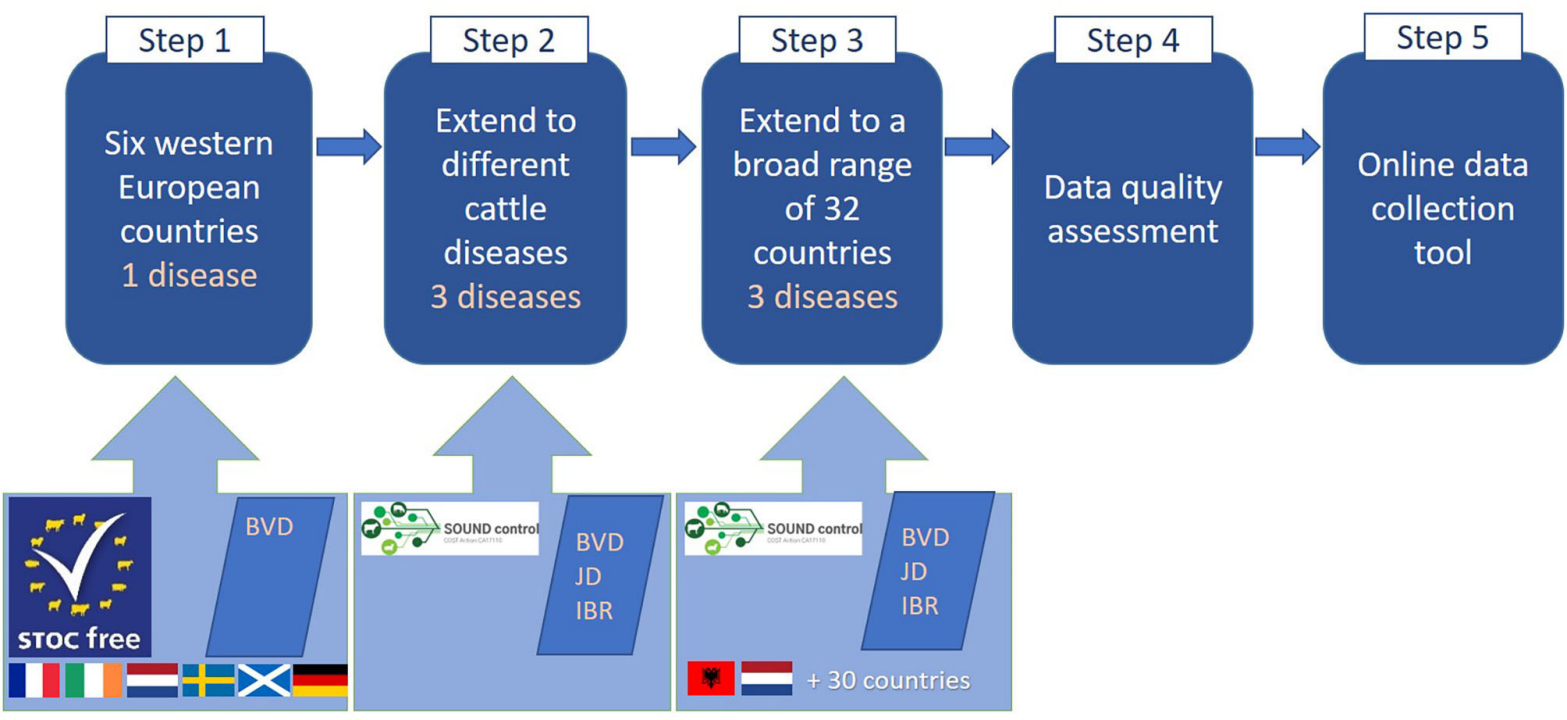
Stepwise process that was followed to come to the final online data collection tool.

A stepwise process was followed to obtain the current version of the online data collection tool (Figure 1). This work was performed within the STOC free and SOUND control project which are summarized in Table 1.

**Table 1:** Overview of the STOC free and SOUND control project.

**Project**	**STOC free**	**SOUND control**
Start date	March 2017	29 October 2018
End date	December 2021	28 October 2022
Number of countries involved	6	32
Geographical scope	Western Europe	Europe
Aim	To develop and validate a new framework (STOC free: Surveillance Tool for Outcome-based Comparison of FREEdom from infection) that enables a transparent and standardized comparison of confidence of freedom for control programmes of both non-regulated and regulated diseases in the EU.	The aim of SOUND control is to coordinate, stimulate, and assist with the initiatives to explore and implement a widely adaptable output-based framework applicable to substantiate the confidence of freedom and cost-effectiveness in current surveillance, control, or eradication programmes for non-regulated cattle diseases in the EU.
More information (progress, news, output)	http://www.stocfree.eu	https://sound-control.eu/

### Step 1: Data Requirements and Availability for Comparison of Freedom From BVDV Infection in Six Western European Countries

A draft data identification tool was developed using Microsoft Excel for BVD in six western European countries (Germany, Ireland, Sweden, Scotland, the Netherlands, and France). In this draft tool, the required aspects that could influence the confidence of freedom from infection in a BVD CP were identified. This tool was based on an earlier study (2) in which the differences between various BVD CPs with respect to freedom of infection for six European countries were identified using the RISKSUR tool (15) as a starting point. The RISKSUR tool was initially developed to build and/or optimize surveillance programmes but this tool has also been used to describe different CPs in a consistent manner (2).

Further work with the tool was conducted by animal health experts from the six afore-mentioned countries, each of whom were partners in the STOC free project (https://www.stocfree.eu/partners). Specifically, information was sought to identify data considered essential for comparison of freedom from BVDV infection, the availability of these data on a quantitative basis, the quality of these data, and the most optimal format of the data. The experts were asked whether the data foreseen to be included in the data collection tool would be available in their country and to evaluate the requested format of all variables and their definitions. Within the tool, there was the possibility to add comments. The experts consulted with other animal health experts in their country when needed, for example when the data were not available at their institute. Before the experts started with their evaluation of the tool, a plenary session was held in which the structure of the tool was explained in detail and they also received this explanation in a separate word file (“Guidelines for the identification and sources of data”: www.stocfree.eu/results/deliverables). Questions that arose during evaluation of the tool could be directed to the developers by email or videocall.

**Figure 2:**
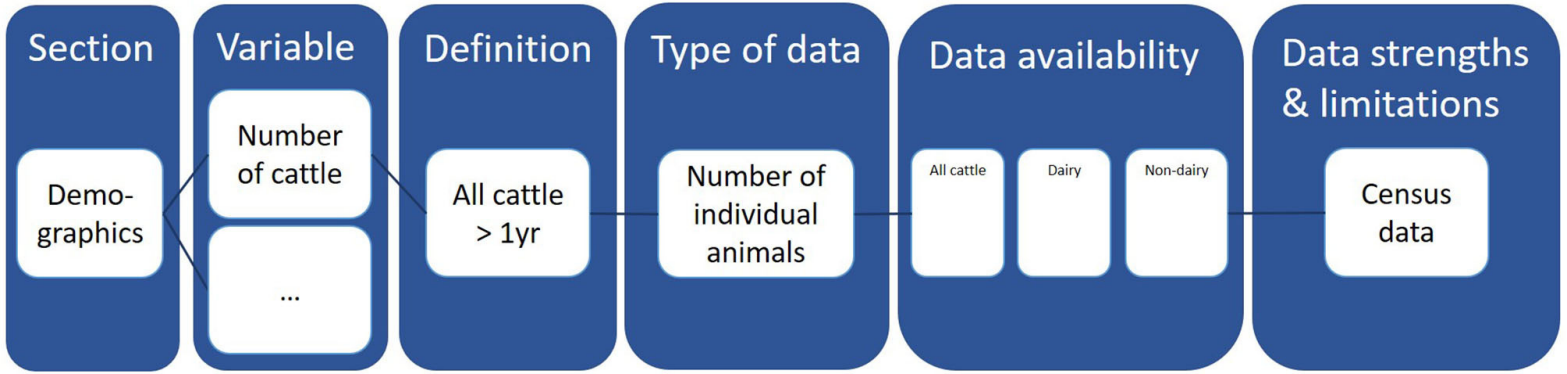
Column headings of the initial Microsoft Excel data collection tool developed for BVD, including an example for the variable “Number of cattle” within the section “Demographics.” The first four columns (section, variable, definition, type of data) are given. Column five “data availability” should be answered with yes/no per group of cattle (all cattle, only dairy cattle, only beef cattle) by the user of the tool. Column six (data strengths and limitations) should also be answered by the user of the tool. An example could be census data.

The tool consisted of three sections addressing cattle demographics, the BVD CP and risk factors for introduction of BVD, respectively. All sections were displayed on one sheet within Microsoft Excel, in the format of a single large table. Each section included all variables for which quantitative data were requested, a definition of the variable, the requested format of the data, and indications of the availability and strengths and limitations of the data (Figure 2). The availability of quantitative data was separated into columns specifying whether the available data included all cattle (dairy and non-dairy) or whether more detailed data on subcategories of cattle were also available: dairy cattle, non-dairy cattle and beef breeding cattle. For BVD it was decided to only include dairy and non-dairy breeding herds (herds where calves are born), given that these populations are considered epidemiologically most relevant for BVD.

### Step 2: Data Requirements When Extending the Tool to Different Cattle Diseases

The tool was subsequently reviewed for possibilities to extend it to other cattle diseases. A different group of experts was involved from the SOUND control project in which more than 100 animal health experts from 32 participating European countries are involved (1, 7). The data collection tool was further extended to JD and IBR in agreement with the animal health experts.

### Step 3: Data Comparability Across a Range of Countries

The next step was to generalize the tool so that it could be applied to all countries throughout Europe. Therefore, the tool was pilot tested by two researchers from two countries with, respectively, developed and developing agricultural sectors i.e., the Netherlands (author ISB) and Albania (author XHK). The results of the pilot test were subsequently presented to 42 animal health experts from 32 different European countries, in a workshop organized for members of the SOUND control consortium. The participants were divided into groups of six people from different countries and were asked to provide feedback on predefined items such as data quality and data availability in their respective countries (Table 2).

**Table 2:** Groups within the SOUND control workshop that discussed specific aspects of the data collection tool.

**Groups**	**Guiding discussion points**
All groups	- Do you understand what data are required? - Do you think the data are available in your country? - Can you say something about the quality of the data? - Do you think all these variables are “MUST KNOW” variables for calculating confidence of freedom? - Do you have any recommendations to improve the tool?
Group 1: Functionality of the tool	- Is it clear how the tool works and what data are required? Are all the variables clear? Do you feel confident about filling in this tool? - What would be a good way to ask about the quality of the data? Keep in mind that it should be objective, comparable between countries and easy to analyze. - Could you provide data for the dairy and beef sector separately? What would be the definitions of dairy and beef in your country?
Group 2 and 3: Demographics	- Do you think that the cut-off value of cattle older than 1 year is satisfactory? Would your country have these data available? Do you think this is the most relevant age group? - Would you be able to answer calving pattern with “yes, seasonal calving”/“no, year-round calving”? Another option for this variable would be to ask for the percentage of calvings in each quarter of the year. Would these data be available in your country? Can you suggest better options?
Group 4 and 5: Control programmes	- How should we define a positive herd or positive animal? This can be different for different diseases and different countries.
Group 6: Test strategies	- Do you think we should ask for the sensitivity and specificity of the tests used in your country? Do you think the data are available? And would you prefer sensitivity and specificity given by the manufacturer or from field studies? We could also include default values for commonly used tests or provide you with ranges of the sensitivity and specificity to choose from. Can you think of any other options?
Group 7 and 8: Risk factors	- Do you think it is important to know how many (pregnant) animals are traded? How would you gather these data? - In many variables we ask you for the percentage of herds, but we give you different options in a drop-down list, including “none,” “0–20,” “20–40” etc. Do you like this or do you prefer exact numbers?

### Step 4: Data Quality Assessment

A data quality evaluation tool was discussed during the above-mentioned SOUND control workshop and developed based on four criteria common in the evaluation of health-related data i.e., accessibility, completeness, accuracy, and timeliness (9, 10). It was envisaged that this tool would enable a standardized and objective evaluation of the quality of each data entry. Within this study, such a tool was developed and incorporated in the data collection tool.

### Step 5: The Online Data Collection Tool

In the final step, the feedback of the workshop was incorporated in a new version of the data collection tool which was subsequently digitalized into an online data collection tool. This was performed with the program Limesurvey (https://www.limesurvey.org/en/). All data entered into the online tool are saved into a database that at this point is only accessible by the authors of this manuscript [Manuscript in preparation: (16)].

## Results

The results section describes the development of the online data collection tool and the key lessons that were learned during this process in three main sections: data requirements for different cattle diseases, data availability and comparability between countries, and data quality.

### Data Requirements for Different Cattle Diseases (BVD, IBR, JD)

The first version of the tool was developed for BVD (“Guidelines for the identification and sources of data”: www.stocfree.eu/results/deliverables). To facilitate inclusion of other cattle diseases, each section (cattle demographics, the BVD CP and risk factors for introduction) was evaluated to ensure that all variables were included that are essential for each of the diseases. No changes were made to the cattle demographics section, as these are similar regardless of the disease evaluated. Small changes were made to the CP section to reflect different test strategies for the different diseases. It was decided to create a single table that can be used for the three selected diseases and, in the future, expand it to all cattle diseases (Table 3). For example, feces and nasal swab samples were not initially included as sample types as these are not regularly used for BVD. However, for JD and IBR, respectively, these samples are also relevant for diagnostic purposes and thus, they should be included in a generalized tool. Also, all variables in the tool include an open answer option which allows for inclusion of answers that were not predefined. The latter is useful when evaluating the completeness of the tool, but in a modeling framework CPs can only be compared using the predefined closed answers. Also, when generalizing the tool to JD and IBR, expansions were made to the risk factor section. Table 4 shows the list of risk factors that were evaluated for inclusion in the tool.

**Table 3:** Test strategy variables with answer options for BVD, JD, and IBR.

**Fields**	**Answer options**
Target group	Older than 2 years, newborn calves, lactating cattle, non-lactating cattle, cattle with clinical signs, purchased cattle, at slaughter, other
Type of sample	Bulk milk, individual milk samples, blood/serum/plasma, tissue (biopsy), tissue (post-mortem), body fluid swabs, fecal smears, feces, environmental samples, slurry
Frequency of testing per year	–
Number of animals tested per test moment	All animals in the target group, representative group of animals (please specify)
Data collection point	Farm, Abattoir, Livestock assembly centers, AI center, Diagnostic laboratory, Market, Other
Collector	Farmer, Veterinarian, Abattoir personnel, other
Test method	Pathogen or antibody detection: ELISA, culture, PCR tests, other
Individual or pooled	Individually tested, Pooled, both possible
If pooled: average number of animals per pool	–

**Table 4:** Risk factors for introduction of infectious cattle diseases that were evaluated for inclusion in the data collection tool.

**Risk factor**
Herd size
Calving pattern
Presence of small ruminants (sheep/goat)
Presence of beef cattle on dairy farms
Introduction of cattle in the herd
Introduction of calves
Introduction of pregnant cattle
Grazing
Communal grazing
Nose to nose contact with cattle from neighboring herds
Contact with wildlife
Farm fragmentation
Natural breeding
Attendance at shows
Housing calves separately from pregnant cattle
Housing calves in individual pens
Sharing transport vehicles between farms
Sharing equipment between farms
Farm clothes for visitors
Compulsory disinfection at entrance
Rodent control
Vector control
Applying manure from other farms on farmland
Feeding colostrum from own dams

### Data Availability and Comparability Across a Range of Countries

To enable application of the tool in all countries throughout Europe, an understanding of data availability and comparability is crucial. When (almost) none of the countries have data available for a variable, the respective variable cannot be used to estimate freedom from infection and thus could not be included in the tool. And when (almost) none of the countries had data available in the requested format, this should be adjusted (e.g., ranges instead of exact numbers).

#### Data Availability Across Six Western European Countries

Data availability in six western European countries (Germany, Ireland, Sweden, Scotland, The Netherlands, and France) was evaluated for all variables included in the first version of the data collection tool developed for BVD. Table 5 shows the availability of quantitative data for some of the variables in the different sections i.e., cattle demographics, CP and risk factors. The first two columns show the requested data in the tool and the remaining part of the table shows a summary of the availability of data as indicated by six countries. As it can be seen in Table 5, most variables related to cattle demographics and the BVD CP are available in (almost) all countries. Very little quantitative data are available for herd-level risk factors such as grazing practices, attendance at cattle shows, vaccination, housing features, and biosecurity practices. More data are available for variables regarding purchase as registration of cattle movements is mandatory in all of the selected countries. The results indicate that for most risk factors no detailed quantitative information is available and thus cannot be included quantitatively in a model.

**Table 5:** Data availability in six European countries for variables on cattle demographics, control programmes and risk factors regarding confidence of freedom from BVDV infection.

**Variable**	**Definition**	**Quantitative (Yes/No)**			
		**All cattle (dairy + non-dairy)**	**Dairy**	**Non Dairy**	**Beef breeding**
**CATTLE DEMOGRAPHICS**					
No. of cattle	Cattle > 1 year	All	NL, IE, SE, FR, UK	NL, IE, SE, FR, UK	NL, IE, SE, FR, UK
No. of cattle herds	Total no. of cattle herds	All	NL, IE, SE, FR, UK	NL, IE, SE, FR, UK	NL, IE, SE, FR, UK
Calving pattern	% of all calvings by month within the past 12 mo.	All	NL, IE, SE, FR, UK	NL, IE, SE, FR, UK	NL, IE, SE, FR, UK
Average no. of births per herd	Within the past 12 mo. per herd	All	NL, IE, SE, FR, UK	NL, IE, SE, FR, UK	NL, IE, SE, FR, UK
Cattle density	No. of cattle per km^2^	All	NL, IE, SE, FR, UK	NL, IE, SE, FR, UK	NL, IE, SE, FR, UK
% of dairy cattle herds with beef cattle on same location	All dairy herds with also beef cattle		IE, SE, FR, UK		
**CONTROL PROGRAMME**					
% of cattle herds participating in CP	% of herds that participate in the CP at the beginning of the year	All	NL, IE, SE, FR, UK	NL, IE, SE, FR, UK	NL, IE, SE, FR, UK
% of animals tested	% of cattle tested for BVD in the territory, during the year	NL, IE, SE, DE, UK	NL, IE, SE, FR, UK	NL, IE, SE, UK	NL, IE, SE, UK
No. of herds that identified one or more PI's.	PI: animal that tested pos. in the initial test or the initial test and re-test, during the year	All	NL, IE, SE, FR, UK	NL, IE, SE, FR, UK	NL, IE, SE, FR, UK
Age at which PI animals were culled	Age at which PI animals were culled during the year	NL, IE, SE, DE, UK	NL, IE, SE, UK	NL, IE, SE, UK	NL, IE, SE, UK
% of free cattle herds	% of cattle herds participating in the CP that have any free status according to the CP, at the beginning of the year	NL, IE, SE, DE, UK	NL, IE, SE, FR, UK	NL, IE, SE, UK	NL, IE, SE, UK
% of free cattle herds that had a breakdown	% of herds participating in CP that had a free status at start of the year but breakdown (ab or virus pos test) during that year.	All	NL, IE, SE, FR, UK	NL, IE, SE, FR, UK	NL, IE, SE, FR, UK
**RISK FACTORS**					
% of cattle herds practicing zero grazing	No grazing during the whole yr	SE	SE	SE	SE
% of cattle herds involved in communal grazing	Grazing animals from different cattle herds together	IE	IE	IE	IE
No. of neighbors at pasture per herd	Pasture where cattle from different herds can have nose to nose contact	NL, SE	NL, SE	NL, SE	NL, SE
% of herds that purchased cattle		All	NL, IE, SE, FR, UK	NL, IE, SE, FR, UK	NL, IE, SE, FR, UK
% of cattle that was purchased from markets/traders	% of purchased cattle	NL, IE, FR, UK	NL, IE, FR, UK	NL, IE, FR, UK	NL, IE, FR, UK
No. of purchase moments in the territory	A purchase event on a specific day to one specific herd	All	NL, IE, SE, FR, UK	NL, IE, SE, FR, UK	NL, IE, SE, FR, UK
% of purchased animals that were pregnant at the moment of purchase		NL, IE, FR	NL, IE, SE, FR	NL, IE, FR	NL, IE, FR
% of herds that quarantine purchased animals that have not been tested before arrival in the herd	% of herds that purchased cattle	FR	FR	FR	FR
% of herds that have animals attending shows		NL, UK	UK	UK	UK
% of herds that vaccinate cattle against BVD		SE, DE	NL, SE	SE	SE
% of cattle herds with goat and/or sheep on same location	Cattle herds with goat and sheep on same location	IE, SE, DE, UK	IE, SE	IE, SE	IE, SE
% of cattle herds that could possibly have contact with wild ruminants	Cattle herds with possible contact with wild ruminants	SE	None	None	None
% of herds that house calves separately from pregnant cattle	% of herds that breed	None	None	None	None
% of herds that share transport vehicles with other cattle herds		None	None	None	None

*NL, The Netherlands; IE, Ireland; SE, Sweden; FR, France; DE, Germany; UK, United Kingdom (here Scotland). Dark green, all six countries have data available. Light green, five countries have data available. Orange, three or four countries have data available. Pink, two countries have data available. Red, at most one country has data available. Gray, not applicable*.

In the workshop, data availability on risk factors for all three infections were discussed. The discussions confirmed that most risk factors are interesting to know but as there is often no data available, or only qualitative data, they probably cannot be included in the data collection tool. At this point, the risk factors considered most important, regardless of data availability, were chosen to be included in the current version of the tool (Appendix 1) to further determine data availability on these risk factors in more different countries. The latter is further studied within SOUND control [Manuscript in preparation: (16)] in a similar way to the initial comparison of six countries (Table 5).

#### Data Comparability in the Netherlands and Albania

To enable comparison of confidence of freedom between countries it is essential that the collected data are comparable. Defining variables in such a way that they cannot be misinterpreted and are workable for different countries within Europe is very challenging. In the first step, the tool was optimized for use in western European countries. For some variables it was impossible to have one definition that fits all countries. As an example, “dairy herds” were variously defined as herds that deliver milk, herds that include a certain percentage of cattle of a dairy breed, herds with newborn calves etc, and “beef herds” could include fattening herds, veal herds, and suckler herds. In this case it was decided that users of the tool should define the population that is covered by their data. For many variables, data were not available at the level of detail requested in the tool e.g., the number of purchased cattle instead of the number of purchased pregnant cattle or the number of cattle per km^2^ land area instead of the number of cattle per km^2^ farm land. For these variables, the definitions were updated into definitions that could be delivered by all countries.

In the next step, the evaluation of the tool for the Netherlands and Albania, showed that both countries are fairly similar in land area, but Albania is more sparsely populated with cattle. The average herd size differs markedly as herds in the Netherlands consist of on average 130 cattle, where the vast majority of herds in Albania consist of <5 animals. An important finding regarding herd size was that the herd size in Albania was registered as the proportion of herds per herd size category and not like in the Netherlands (and most other countries in western Europe) where for each herd the exact number of animals is known. Therefore, the data collection tool was adapted and requests the percentage of herds per herd size category as this could be delivered by both countries. This highlights that cattle demographics can be very different between countries and knowledge of the extremes is needed to decide how to define and structure data requests in a data collection tool. Disease control and monitoring is further developed in the Netherlands compared to Albania. In the Netherlands, there are many CPs, both compulsory and voluntary, but in Albania there are only a few voluntary CPs. Also, large volumes of high quality data are collected routinely in the Netherlands, whereas there is only limited quantitative data available in Albania. However, semi-quantitative or qualitative data was often available, which could be facilitated in a data collection tool. For example, it is not exactly known how many cattle farms purchased cattle, but experts could give an estimate. This shows the need of including a data quality assessment tool within the data collection tool and including uncertainty in an output-based framework.

### Assessment of Data Quality

The needs of a data quality assessment tool were discussed during the workshop. All participants agreed that an objective assessment of data quality is essential to compare the confidence in the probability of freedom. Aspects that were considered important were data sources and accessibility, completeness of data, timeliness of data, and data accuracy. These aspects were incorporated in a data quality evaluation tool (Table 6). For each variable, the participant is asked to score each of these criteria with a score from 1 to 3, meaning poor, fair, good. To ensure objectivity in this scoring, the meaning of each score for each criterion is described in Table 6.

**Table 6:** Data quality evaluation tool.

Evaluation\Quality criteria	**Accessibility**	**Completeness**	**Timeliness**	**Accuracy**
POOR score−1	The variable is not routinely collected **AND** you only have access to this information via indirect sources (e.g., research studies)	The variable is not mandatory to enter in the database **AND** completeness of data is unknown **OR** lower than 80%	It is unknown when data is updated	The variable is entered manually to the dataset **AND** No data validation is performed (e.g., the data are not used for any other purpose).
FAIR Score−2	The variable is not readily available but can be obtained by combining multiple sources **AND/OR** data is available, but access is associated with fee/approval of data-owner	The variable is not mandatory to enter in the database **AND** completeness of data set is >80%	The data are updated once or twice per year	The variable is entered manually **AND** data validation procedure is sometimes implemented (e.g., variable is used on a regular basis for creating reports, or combined with other data sources)
GOOD Score−3	The variable is obtained from one data source **AND** can be extracted when needed	The variable is mandatory to enter in the database **OR** The variable is not mandatory to report, **AND** completeness of data set is close to 100%	The data are updated real time	The variable is collected and entered by an automatic system/robot **OR** The variable is entered manually **AND** data validation procedure is always implemented (e.g., variable is used on a regular basis for creating reports, or combined with other data sources)

The overall data quality is calculated per variable by adding up the individual scores for accessibility, completeness, timeliness and accuracy. The four criteria are equally weighted, but the individual scores per criterion are also available e.g., evaluation of accessibility of all cattle demographic data. The quality score can be used to evaluate comparability of data quality between countries.

### The Online Data Collection Tool

**Figure 3:**
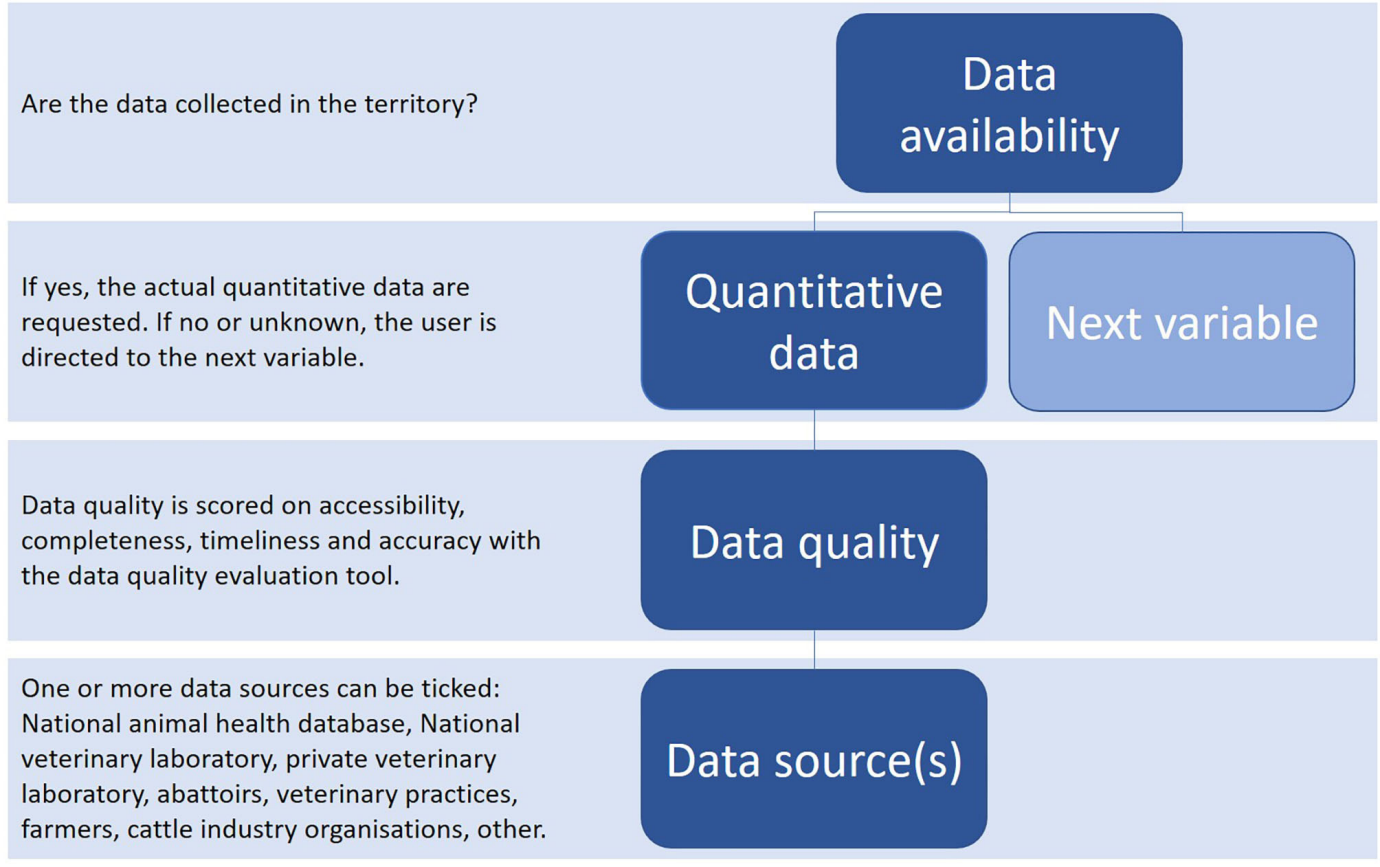
Schematic overview of the question structure of the online data collection tool. For each variable within the data collection tool this structure is followed from top to bottom.

**Figure 4:**
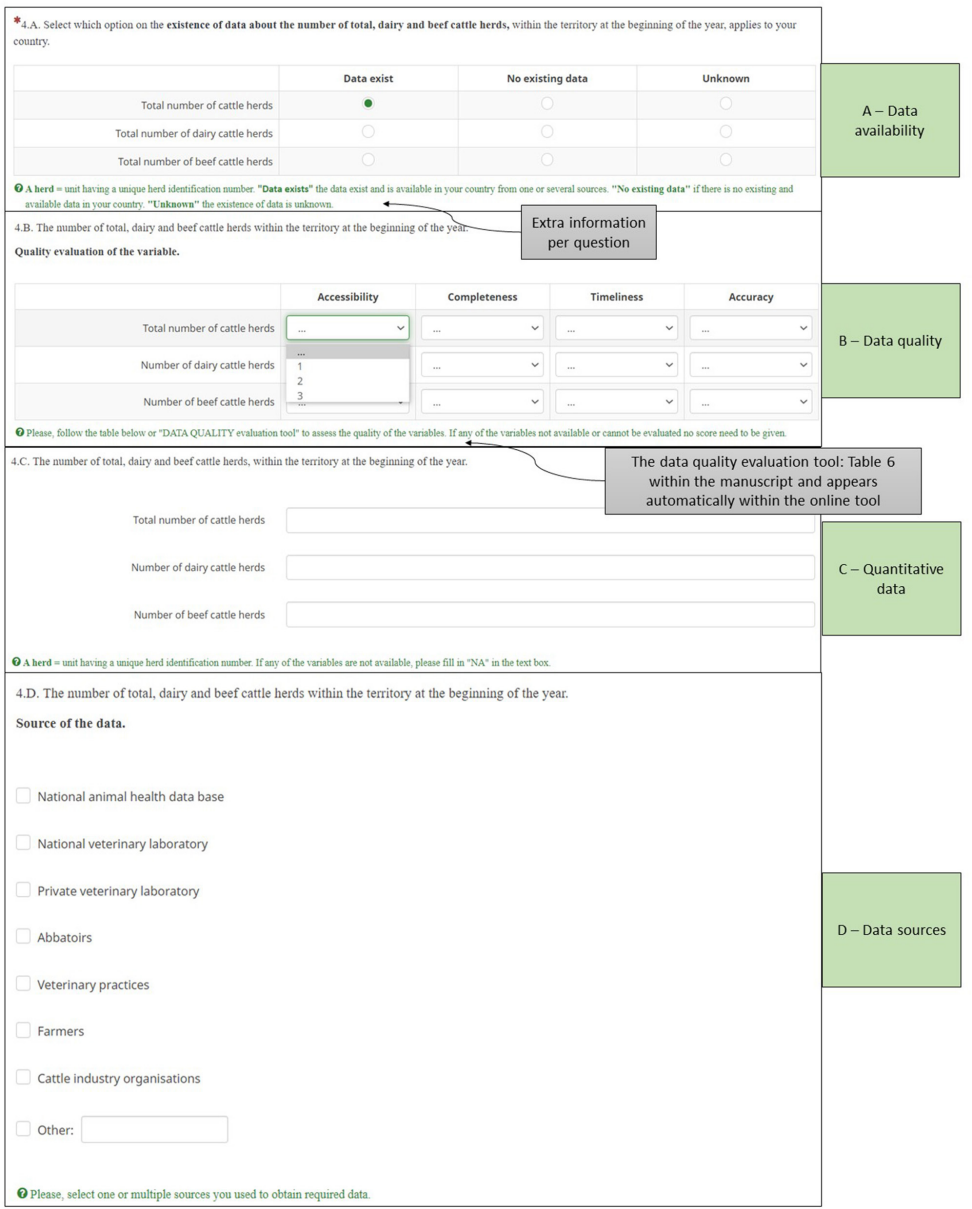
Format of each question within the online data collection tool.

The current version of the tool is available online through Limesurvey only for testing purposes by the COST participant countries (https://sound-control.eu/). The online tool includes some general participant information and three main sections that need to be filled: cattle demographics, risk factors and disease CPs. The cattle demographics section includes 11 variables, the risk factors section 18 variables and the disease CPs section 8 variables and a separate section about the test strategy per target group of animals tested within the CP. The CP section includes JD, IBR, and BVD. All variables and the format of the requested data that are included in the tool can be found in Appendix 1. The focus of the tool is on data availability, data quality and data sources (Figure 3). Each question in the tool is structured in the same way to make it easy to fill (Figure 4). Any additional explanation that was made available before in a separate word file, is now included per question in green text. Depending on the availability and accessibility of data it may take 4–5 h to fill in the tool.

## Discussion

The data collection tool was developed to collect data required for an output-based framework for estimation of freedom of infection for a range of cattle diseases and countries within Europe. In this paper, we presented the key learnings from the development process of the data collection tool from the beginning, when it was built for a single disease and six countries, to an online tool that can be applied to multiple cattle diseases and for a large number of countries.

The tool was developed to be self-explanatory and easy to use. Depending on the number of different CPs for which the user wants to use the tool, the amount of work can be substantial. However, the demographics and risk factors section will be similar regardless of the disease within a country and therefore, only needs to be filled in once. Additionally, within a country many of the demographic parameters are already known and data is readily available. When this tool is incorporated in a modeling framework to actually calculate the confidence of freedom, data can be saved and can be easily changed or supplemented when there are changes in the cattle demographics, CPs or risk factors.

The results indicate that extending the data collection tool to different cattle diseases is achievable. At most, the cattle population of interest could differ e.g., different age groups or production types. Also, the variables regarding the CPs do not differ substantially between diseases, being mainly a matter of including a wide range of answer options in, for example, the test strategy. The risk factor part could vary, however the most important risk factors, such as cattle movements and direct and indirect contact between animals originating from different herds, are relevant for all infectious cattle diseases.

The biggest challenge was to request data in such a way that the tool could be filled in by experts from different European countries. The partners agreed with the initial version of the tool but when people actually filled the tool they encountered unforeseen difficulties, e.g., the definition was not as clear as thought, the data were not available, data were available but in a different format, data were not accessible or people felt that the entered data needed additional explanation. Therefore, it is extremely important to clearly define the variables to ensure that users understand what data should be delivered, why the specific format is requested and to have pilot test runs in which the tool actually has to be filled. To obtain a broad overview of the data availability and format in many different countries, international collaboration in projects such as STOC free and SOUND control was crucial. In a follow up study, partners from all countries involved within SOUND control were asked to fill in the tool for their country. The results of this study can be used to further optimize the online data collection tool and to decide on how to change the online tool into a publicly available tool (16). After the tool is finalized the SOUND control consortium has to discuss on the maintenance and sustainability of the tool. The tool will be made available on the SOUND control website and will be kept up to date throughout the SOUND control project. The website will remain available after the end of the project. For sustainability, the tool will be advertised to EFSA and European stakeholder organizations such as FESASS (The European Federation for Animal Health and Sanitary Security), to show the merit of keeping the tool up to date. The plan on maintenance and sustainability is still under discussion within work group 2 “Data requirements and availability” of the SOUND control project (https://sound-control.eu/about/wg/wg2/).

For some variables, such as the number of dairy and beef cattle, standardization was neither possible nor desired because an output-based framework should be flexible and each CP is set to the country-specific definitions. For these variables, each country's definition should be captured, which should in this case be the population covered by the CP. Seemingly easy to collect data on variables, such as herd size, were more difficult to query for inclusion in an output-based framework than expected. For example, in this case, some countries only count adult cattle while other countries also include calves in this number. And even with only asking for the number of adult cattle, comparison can be problematic because in some countries cattle are counted as adult at 1 year of age compared to 2 years of age or from the moment their first calf is born in other countries. Therefore, we evaluated for each variable whether standardization was desired and then whether the format of data could be delivered by all countries. In the example of the variable “cattle density,” a definition of the number of cattle per km^2^ in the country was agreed. However, some countries can provide more detailed data at regional level in their country. Such detailed information provides the opportunity to distinguish low cattle density areas from high cattle density areas and their respective risks. Another disadvantage of the applied definition was that it did not correct for land area less suitable or not used for cattle farming e.g., mountainous or urban areas. Nevertheless, the chosen definition could be calculated for each country in a similar way which enabled comparison of the value of this variable between countries.

Another challenge was to find a balance between the amount of detail that could potentially be sought and what was actually needed. Up to this point, the inclusion of variables was mainly driven by the availability of data, while the data collection tool is intended to be linked to an output-based model. For the latter, only data should be requested that is needed to populate the model to calculate freedom from infection for different cattle diseases in different countries. At present, there is a first version of an output-based model available for BVD, the STOC free model (17), which is a Bayesian Hidden Markov model that incorporates test results and risk factors. The model performance was evaluated for BVD control programmes in six European countries. The current version of the data collection tool requests a lot of data to obtain a complete overview of the cattle demographics, the CPs and risk factors in a country. However, the STOC free model only incorporates a limited number of these parameters when generating an output. Consideration should be given to the added value of including an extra variable within the model. Herd-level risk factor information such as the possibility of nose-to-nose contact between herds, herds attending cattle shows, the use of quarantine facilities etc. are of epidemiological interest at herd-level but may not have major influence on the confidence of freedom at country level, and would substantially complicate the model. Even where they are deemed important, their incorporation is constrained because in most countries only an approximation can be given for these variables. Therefore, it seems challenging to include most of the risk factors. One of the questions that was raised during this study was whether qualitative data should be collected with the data collection tool when no quantitative data were available, with this being particularly relevant for many of the risk factors. Within the data collection tool, this could be facilitated together with the quality assessment tool. However, this requires further study to determine whether this is useful in the context of assessing confidence of freedom through an output-based model. The data collection tool can be further improved in an iterative process at the same time as model development. This would apply to the STOC free model, but also to any other output-based model that might subsequently be developed for estimating the confidence of freedom.

The current data collection tool requests data about cattle demographics, CP test results and risk factors. Other aspects that could influence confidence of freedom calculations include biosecurity measures and socioeconomic considerations, however, these are not currently included in the model. Currently, limited data are available to accurately quantify the concept of biosecurity. As one example, the quarantine of purchased animals could be effective means to prevent introduction of infection in the herd, but to obtain reliable data on this is very difficult. The same challenges apply with respect to data on hygiene measures, grazing practices, housing practices etc. For socioeconomic aspects, such as farmer behavior and farm costs, more research is needed into which aspects are important and how these could be incorporated in an output-based framework. Further work on this is currently performed in the SOUND control project.

The data collection tool was developed to collect data for three relevant cattle diseases in a wide range of countries within Europe as input for output-based methods to calculate freedom of infection. In this study, we can conclude that the initial seemingly easy task of development of a data collection tool was far more complex than foreseen. Key aspects that need to be considered in such a tool are alignment and clarification of variable definitions, data availability, a clear distinction between data essential for comparison of freedom of infection vs. data that are interesting to know, and an objective means for data quality assessment. These key learnings can support studies in which data on infectious diseases in livestock from different countries should be collected to compare freedom of infection.

## Data Availability Statement

The datasets presented in this study can be found in online repositories. The names of the repository/repositories and accession number(s) can be found at: https://www.stocfree.eu/results/deliverables.

## Author Contributions

AR prepared the manuscript. All authors contributed to the conceptualization and revising the manuscript.

## Conflict of Interest

The authors declare that the research was conducted in the absence of any commercial or financial relationships that could be construed as a potential conflict of interest.
