# Supplementary material for: Key Learnings During the Development of a Generic Data Collection Tool to Support Assessment of Freedom of Infection in Cattle Herds
Source: Front Vet Sci. 2021 Apr 26;8:656336. doi: 10.3389/fvets.2021.656336 (PMC8107354; doi:10.3389/fvets.2021.656336)
Supplement: Supplementary file 1 [file Table_1.DOCX]

**Appendices**

**Appendix 1: Variables included in the current online data collection tool**

| **Section** | **Variables^1^** | **Definition** |
| --- | --- | --- |
| General | Date of filling in the table | DD/MM/YYYY |
| information | Year represented by the data | DD/MM/YYYY |
|  | Country | Country or region covered by the data |
|  | Contact information | (optional) |
|  | Definitions of dairy and beef cattle in the respective country | Dairy and beef cattle as covered by the data |
| Demographics^2^ | Number of cattle older than 1 year |  |
|  | Number of cattle herds | Units with a unique identification number |
|  | Average number of cattle older than 1 year per herd |  |
|  | Percentage of herds per herd size category | The categories can be indicated by the user, e.g. <10, 10-50, 51-100, 101-500, >500 |
|  | Number of calvings | In one year in the territory |
|  | Number of herds where calves are born |  |
|  | Average number of calvings per herd |  |
|  | Presence of seasonal calving pattern | Seasonal calving: 2/3 of the calves born during a 2-3 month period. |
|  | Cattle density | Number of animals in total land area of the territory (animals/km^2^) |
|  | Number of herds that also have sheep/goats |  |
|  | Number of mixed farms | All dairy herds that also have a type of beef cattle such as veal calf, suckler cattle etc |
| Risk factors^3^ | Number of herds that purchased cattle | Introduction of cattle in the herd or territory originating from outside the territory |
|  | Number of cattle that was purchased |  |
|  | Number of cattle that were calves at the time of purchase | Calves are animals in their first year |
|  | Number of cattle that were pregnant at the time of purchase | Cattle that delivered a calf within nine months after purchase |
|  | Number of herds that practice zero grazing | No grazing during the whole year |
|  | Number of herds that are involved in communal grazing | Grazing animals from different cattle herds together |
|  | Number of herds that are fragmented | Farms where two or more geographically separated tracts of lands are operated |
|  | Number of herds that apply natural breeding | Herds that own or use a bull for breeding |
|  | Number of herds that participate in shows |  |
|  | Number of herds that house calves separately from pregnant cattle |  |
|  | Number of herds that house calves in individual pens |  |
|  | Number of herds that share transport vehicles with other herds |  |
|  | Number of herds that share equipment with other herds |  |
|  | Number of herds that provide clothing for visitors |  |
|  | Number of herds that apply compulsory disinfection procedures |  |
|  | Number of herds that apply rodent control programs |  |
|  | Number of herds that apply vector control programs |  |
|  | Number of herds that apply manure from other herds on their land |  |
| Disease control programs^4^ | Number of herds participating in the control program |  |
|  | Number of herds tested for the disease |  |
|  | Animal and herd level prevalence of the disease | Based on the total number of animals and herds present in the territory |
|  | Number of herds participating in the control program that have the disease-free status |  |
|  | Number of herds participating in the control program that identified one or more disease positive animals |  |
|  | Number of cattle participating in the control program that was disease positive |  |
|  | Presence of a culling policy as part of the control program | Regulations about culling of animals tested positive for the disease (e.g. herds cannot export animals). |
|  | Is vaccination for the disease allowed? |  |
|  | Test strategy: target group, type of sample, frequency of testing, number of animals tested per test moment, data collection point, collector, test method, individual or pooled and average number of animals per pool | Test strategy per target group. Target groups are groups of cattle that are tested within the control program |

^1^ For all variables where numbers are requested, percentages could be also provided. ^2^ Data requested refer to dairy and beef cattle together (total cattle) and also separately if available.

^3^ Even though the results of the six countries showed that some of these risk factors could not be included because there were no data available, they were included in the current version of the tool to further test availability of data in more countries.

^4^ The table can be filled in for Johne’s disease (JD), Infectious Bovine Rhinotracheitis (IBR) and Bovine Viral diarrhea (BVD).
